# Supplementary material for: Health literacy, cognitive ability and smoking: a cross-sectional analysis of the English Longitudinal Study of Ageing
Source: BMJ Open. 2018 Oct 27;8(10):e023929. doi: 10.1136/bmjopen-2018-023929 (PMC6224719; doi:10.1136/bmjopen-2018-023929)
Supplement: Author's manuscript [file bmjopen-2018-023929.draft_revisions.pdf]

BMJ Open is committed to open peer review. As part of this commitment we make the peer review history of every article we publish publicly available.

When an article is published we post the peer reviewers' comments and the authors' responses online. We also post the versions of the paper that were used during peer review. These are the versions that the peer review comments apply to.

The versions of the paper that follow are the versions that were submitted during the peer review process. They are not the versions of record or the final published versions. They should not be cited or distributed as the published version of this manuscript.

BMJ Open is an open access journal and the full, final, typeset and author-corrected version of record of the manuscript is available on our site with no access controls, subscription charges or pay-per-view fees (http://bmjopen.bmj.com).

If you have any questions on BMJ Open's open peer review process please email info.bmjopen@bmj.com

BMJ Open

The role of health literacy and cognitive ability in smoking: findings from the English Longitudinal Study of Ageing

Journal:	*BMJ Open*
Manuscript ID	bmjopen-2018-023929
Article Type:	Research
Date Submitted by the Author:	07-May-2018
Complete List of Authors:	Fawns-Ritchie, Chloe; University of Edinburgh, Centre for Cognitive Ageing and Cognitive Epidemiology<br>Starr, John; University of Edinburgh<br>Deary, Ian; University of Edinburgh, Centre for Cognitive Ageing and Cognitive Epidemiology, Department of Psychology
Keywords:	EPIDEMIOLOGY, STATISTICS & RESEARCH METHODS, PUBLIC HEALTH

SCHOLARONE™ Manuscripts

For peer review only

The role of health literacy and cognitive ability in smoking: findings from the English Longitudinal Study of Ageing

Chloe Fawns-Ritchie[1,2], John M Starr[1,3], and Ian J Deary[1,2*]

[1]Centre for Cognitive Ageing and Cognitive Epidemiology, University of Edinburgh, Edinburgh, UK

[2]Department of Psychology, University of Edinburgh, Edinburgh, UK

[3]Alzheimer Scotland Dementia Research Centre, University of Edinburgh, Edinburgh, UK

*Corresponding author: Ian J Deary, Centre for Cognitive Ageing and Cognitive Epidemiology, Department of Psychology, University of Edinburgh

7 George Square

Edinburgh, EH8 9JZ

Scotland, UK

Telephone: +44 131 650 3452

ian.deary@ed.ac.uk

Word count: 2,893

For peer review only

For peer review only - http://bmjopen.bmj.com/site/about/guidelines.xhtml

ABSTRACT

Objectives We used logistic regression to investigate whether health literacy and cognitive ability independently predicted whether participants have ever smoked and, in ever smokers, whether participants still smoked nowadays.

Design Cross-sectional study.

Setting This study used data from the English Longitudinal Study of Ageing, which is a cohort study of adults aged 50 years and older living in England.

Participants 8,734 (mean age=65.31 years, SD=10.18) English Longitudinal Study of Ageing participants who answered questions about their current and past smoking status, and completed cognitive ability and health literacy tests at Wave 2.

Primary and secondary outcome measures The primary outcome measures were whether participants reported ever smoking at Wave 2 and whether ever smokers reported still smoking at Wave 2.

Results In age- and sex-adjusted models, limited compared to adequate health literacy was associated with greater odds of reporting ever smoking (OR=1.133, 95% CI 1.025 to 1.252). A 1SD higher general cognitive ability score was associated with reduced odds of reporting ever smoking (OR=0.934, 95% CI 0.886 to 0.986). The associations were non-significant after adjusting for education and occupational class. In ever smokers, limited compared to adequate health literacy was associated with greater odds of being a current smoker (OR=1.312, 95% CI 1.143 to 1.506). A 1SD higher general cognitive ability score was associated with reduced odds of being a current smoker (OR=0.771, 95% CI 0.715 to 0.832). These associations remained significant, though somewhat attenuated, after adjusting for education and occupational class.

For peer review only - http://bmjopen.bmj.com/site/about/guidelines.xhtml

Conclusions This study found that health literacy and cognitive ability were independently associated with whether participants started smoking and whether ever smokers continued to smoke.

For peer review only

For peer review only - http://bmjopen.bmj.com/site/about/guidelines.xhtml

Strengths and limitations of this study

- This study used a large sample (n = 8,734) from the English Longitudinal Study of Ageing; a study designed to be representative of the English population aged over 50 years.
- This analysis was cross-sectional and therefore cannot determine the direction of the association between smoking, health literacy and cognitive ability.
- This study included measures of both health literacy and cognitive ability which allowed us to investigate whether health literacy was associated with smoking status when controlling for cognitive ability.
- Smoking status was self-reported.

For peer review only

INTRODUCTION

The effects of smoking on ill-health have been known for decades. The prevalence of smoking in the UK is falling and the number of smokers who are quitting is increasing.[1] Despite this, nearly 16% of the UK population were current smokers in 2016[1] and smoking remains one the largest causes of preventable morbidity and mortality in the UK.[1, 2] Understanding the characteristics of individuals who take up smoking, and who quit smoking is important to be able to design and target smoking education and interventions.

Cognitive ability is associated with smoking. Individuals who smoke have lower scores on cognitive tests than those who have never smoked.[3-5] Smokers show steeper ageing-related cognitive decline[4-7] and have increased risk of Alzheimer's disease,[6, 8] compared to non-smokers. One possible pathway between smoking and cognitive ability is that smoking has harmful consequences for the vascular system, which could in turn affect cognitive functioning.[6, 9]

A perhaps complementary explanation is that individuals who have lower cognitive ability in youth are more likely to take up smoking and less likely to quit.[9-11] Corley et al.[9] found that, when controlling for childhood cognitive ability, the association between smoking and cognitive function in old age was attenuated, and in some cases, became non-significant. Two studies[10, 12] found different patterns when investigating the relationship between childhood cognitive ability and reporting ever smoking. One study using the 1970 British Birth Cohort[10] found that individuals with higher childhood cognitive ability were less likely to have ever smoked in a sample of middle-aged participants, whereas another report, based on two of the Midspan prospective cohort studies[12], found no association between cognitive ability in childhood and ever smoking in a sample of older adults. Both these

For peer review only

For peer review only - http://bmjopen.bmj.com/site/about/guidelines.xhtml

studies, however, found that among ever smokers, individuals with higher childhood cognitive ability were more likely to quit smoking.[10, 12]

A person's health literacy may also play a role in smoking status, though the evidence for an association between health literacy and smoking is mixed.[13-16] Health literacy is the capacity to acquire, process and use health information to successfully navigate all aspects of health, including the ability to use health documents, interact with health care professionals, and undertake health promoting behaviours to prevent future ill-health.[17, 18] Some studies have found that individuals with lower health literacy are more likely to smoke,[13, 14] whereas others have not.[15, 16] It is possible that individuals who have limited health literacy are less aware of the adverse effects of smoking on health, and may be less able to understand and utilise smoking cessation services.

The current study sought to determine whether health literacy and cognitive ability, when studied together, have independent associations with smoking. Drawing on the English Longitudinal Study of Ageing (ELSA), we first investigated whether health literacy and cognitive ability were independently associated with whether individuals had ever smoked. Second, we investigated whether there was a relationship between health literacy, cognitive ability and whether ever smokers continued to smoke, or quit.

METHODS

Participants

This study uses data from Wave 2 of the ELSA study, a cohort study of individuals aged 50 years and older living in England.[19] A total of 11,391 participants took part in Wave 1 in 2002-03, and these participants have been followed up every two years. More information on this cohort is provided elsewhere.[19] The current sample consists of participants who

For peer review only - http://bmjopen.bmj.com/site/about/guidelines.xhtml

completed the Wave 2 interview (n=8,780); this is the first wave in which health literacy was assessed. Ethical approval was granted from the National Research and Ethics Committee. This study conformed to the principles embodied in the Declaration of Helsinki.

Measures

Smoking

Two aspects of smoking status (ever versus never smoker and current versus former smoker) were the outcome variables in these analyses. Participants were asked 'Have you ever smoked cigarettes?'. Participants were categorised as ever smokers if they answered 'yes' and never smokers if they answered 'no' at either Wave 1 or 2. Ever smokers were additionally asked 'Do you smoke cigarettes at all nowadays?'. Ever smokers who answered 'yes' to smoking cigarettes nowadays at Wave 2 were categorised as current smokers, whereas ever smokers who answered 'no' were categorised as former smokers.

Health literacy

Health literacy was assessed at Wave 2 using a four-item comprehension test previously used in the International Adult Literacy Survey.[20] Participants were presented with a piece of paper containing instructions similar to those that would be found on a packet of over-the-counter medication. Participants were instructed to read the medicine label and were then asked four questions about the information on this label (e.g., 'what is the maximum number of days you may take this medicine?'). The label was available to the participant to refer to at any time. This task was designed to measure the skills thought to be required to understand and use health materials correctly, such as the ability to read and use numbers in a health context.[21] One point was awarded for each correctly answered question (range 0-4). As has

For peer review only

been done in previous ELSA studies[22, 23] health literacy scores were categorised as 'adequate' (4/4 correct) or 'limited' (≤ 3 correct).

Cognitive function

Four tests of cognitive function that were administered at Wave 2 of the ELSA study were used here. These tests are thought to assess episodic memory, executive function and processing speed; these are cognitive domains which tend to decline on average with increasing age.[24, 25] In the word list recall test, participants heard a list of 10 words which they had to recall immediately (immediate recall test) and again after a short delay (delayed recall test). The score on each occasion was the number of words remembered (range 0-10). Executive function was assessed using categorical verbal fluency (number of animals named in 60 seconds). The letter cancellation test, in which participants were to scan rows of letters and score out all P's and W's, was used to measure processing speed. The score is the number of P's and W's scored out in 60 seconds. Scores on the four cognitive tests were entered into a principal components analysis. Prior to this, individuals who scored 0 or greater than 4 SD above the mean on the animal fluency test and the letter cancellation test were removed. Scores of 0 indicate that the participant did not understand the task, and scores 4 SD above the mean were seen as dubiously high given the one minute time limit for these tests. Only the first component had an eigenvalue greater than one and the scree plot indicated only one component. Scores on the first unrotated principal component were used as a measure of general cognitive ability. This component accounted for 56% of the total variance in the four cognitive tests. The loadings of the tests were: immediate word recall=0.83; delayed word recall=0.84; animal naming=0.70; letter cancellation=0.59.

Covariates

For peer review only - http://bmjopen.bmj.com/site/about/guidelines.xhtml

Age in years, sex, age of leaving full-time education and occupational social class were used as covariates. For confidentially reasons, owing to there being few of them, participants aged over 90 years have had their age set to 90. Participants were asked at what age they left continuous full-time education (recorded as not yet finished, never went to school, 14 or under, at 15, at 16, at 17, at 18, and 19 or over). For the purpose of this study, age of leaving full-time education was categorised as 14 years or under, 15-16 years, 17-18 years, and 19 years or over. Occupational social class was categorised using the National Statistics Socio-economic classification (NS-SEC) 3-cateogries: managerial and professional, intermediate, and routine and manual.[26]

Patient and public involvement

Participants were not involved in the development of any part of this study.

Statistical analysis

Two sets of analyses were carried out. First, ever smokers were compared with never smokers; second, current smokers were compared with former smokers. To determine whether ever versus never smokers, and current versus former smokers differ on health literacy, general cognitive ability, and sociodemographic variables, t-tests were used for normally-distributed continuous variables, Mann-Whitney U tests were used for non-normal continuous variables, and χ^2 tests were used for categorical variables. Binary logistic regression was used to examine the independent associations of health literacy and general cognitive ability on smoking status. Age and sex were entered in all models. Health literacy and general cognitive ability were entered individually in models 1 and 2, respectively. To determine whether both health literacy and general cognitive ability are independently

For peer review only - http://bmjopen.bmj.com/site/about/guidelines.xhtml

associated with smoking, both predictors were included in model 3. Model 4 additionally adjusted for age of finishing full-time education and occupational social class to determine whether any associations between health literacy, cognitive function and smoking remained after controlling for these socioeconomic variables.

RESULTS

Of the 8,780 participants who completed the wave 2 interview, 8,734 participants had complete data on smoking, cognitive ability, and health literacy, and they make up the analytic sample. Participant characteristics are reported in Table 1. A total of 5,525 (63.3%) of the sample reported ever smoking, whereas 3,209 (36.7%) participants reported having never smoked. Ever smokers were more likely to have limited health literacy and had lower general cognitive ability than never smokers. Ever smokers were older, were more likely to be male, have left full-time education at a younger age, and have a lower occupational social class than never smokers. A total of 1,356 (15.5%) reported that they still smoked cigarettes at Wave 2, whereas 4,169 (47.7%) participants reported that they had stopped. Current smokers were more likely to have limited health literacy than former smokers; however, the two groups did not differ on general cognitive ability. Current smokers were younger, more likely to be female, have left full-time education at a younger age, and to have a lower occupational social class than former smokers. Given that current smokers were, on average, 4.5 years younger than former smokers, we tested the point-biseral correlation between smoking status and general cognitive ability, with and without controlling for age. When not controlling for age, the correlation between smoking and general cognitive ability was 0.004 (p=0.742). Adjusting for age, the correlation was -0.100 and this was significant (p<0.001).

For peer review only - http://bmjopen.bmj.com/site/about/guidelines.xhtml

Table 1: Participant characteristics according to smoking status (n = 8,734)*

	Smoking history			Smoking cessation†		
	Ever smoker (n = 5,525)	Never smoker (n = 3,209)	P-value for difference	Current smoker (n = 1,356)	Former smoker (n = 4,169)	P value for difference
Health literacy, n (%)			0.001			< 0.001
Adequate	3,647 (66.0)	2,233 (69.6)		840 (61.9)	2,807 (67.3)	
Limited	1,878 (34.0)	976 (30.4)		516 (38.1)	1,362 (32.7)	
General cognitive ability, mean (SD)	-0.05 (1.00)	0.08 (0.99)	< 0.001	-0.04 (0.99)	-0.05 (1.01)	0.739
Age (years), mean (SD)	65.53 (10.13)	64.93 (10.24)	0.005	62.12 (9.12)	66.64 (10.20)	< 0.001
Sex, n (%)			0.001			< 0.001
Female	2,752 (49.8)	2,172 (67.7)		761 (56.1)	1,991 (47.8)	
Male	2,773 (50.2)	1,037 (32.3)		595 (43.9)	2,178 (52.2)	
Age left full-time education, n (%)			< 0.001			< 0.001
14 years or under	1,104 (20.6)	553 (17.6)		233 (17.7)	871 (21.5)	
15-16 years	2,936 (54.8)	1,578 (50.2)		856 (65.0)	2,080 (51.4)	
17-18 years	665 (12.4)	488 (15.5)		128 (9.7)	537 (13.3)	

For peer review only - http://bmjopen.bmj.com/site/about/guidelines.xhtml

19 years or over	657 (12.3)	526 (16.7)		99 (7.5)	558 (13.8)	
Occupational social class, n (%)			< 0.001			< 0.001
Managerial & professional	1,677 (30.8)	1,047 (33.2)		274 (20.6)	1,403 (34.2)	
Intermediate	1,263 (23.2)	884 (28.1)		312 (23.4)	951 (23.2)	
Routine & manual	2,499 (45.9)	1,218 (38.7)		747 (56.0)	1,752 (42.7)	

*Characteristics for age left full-time education are based on a subset of 8,507 participants with this data and characteristics for occupational social class are based on a subset of 8,588 participants with this data.

†For smoking cessation comparisons, the ever smoker category is divided into whether ever smokers are current or former smokers.

For peer review only - http://bmjopen.bmj.com/site/about/guidelines.xhtml

Table 2: Odds ratios and 95% CIs from logistic regression models of whether participants have ever smoked

	Model 1 (n = 8,734)<br>OR (95% CI)	Model 2 (n = 8,734)<br>OR (95% CI)	Model 3 (n = 8,734)<br>OR (95% CI)	Model 4 (n = 8,367)<br>OR (95% CI)
Health literacy				
Adequate	Reference	Reference	Reference	Reference
Limited	1.174 (1.067 to 1.293)**	-	1.133 (1.025 to 1.252)*	1.097 (0.989 to 1.218)
General cognitive ability†	-	0.917 (0.871 to 0.965)***	0.934 (0.886 to 0.986)*	1.003 (0.947 to 1.062)
Age (years)	1.004 (1.000 to 1.008)	1.001 (0.996 to 1.006)	1.001 (0.996 to 1.006)	1.002 (0.996 to 1.007)
Sex				
Female	Reference	Reference	Reference	Reference
Male	2.112 (1.929 to 2.313)***	2.077 (1.896 to 2.276)***	2.087 (1.905 to 2.287)***	2.152 (1.956 to 2.367)***
Age left full-time education				
14 years or under	-	-	-	Reference
15-16 years	-	-	-	1.015 (0.880 to 1.171)
17-18 years	-	-	-	0.828 (0.690 to 0.993)*
19 years or older	-	-	-	0.692 (0.571 to 0.839)***

For peer review only - http://bmjopen.bmj.com/site/about/guidelines.xhtml

Occupational class				
Managerial & professional	-	-	-	Reference
Intermediate	-	-	-	0.919 (0.811 to 1.042)
Routine & manual	-	-	-	1.206 (1.067 to 1.362)**

*p<0.05, **p<0.01, *** p<0.001.

†ORs (95% CIs) for general cognitive ability are the odds of reporting ever smoking for a 1 SD increase in general cognitive ability.

For peer review only - http://bmjopen.bmj.com/site/about/guidelines.xhtml

Table 3: Odds ratios and 95% CIs from logistic regression models of whether ever smokers still smoke nowadays

	Model 1 (n = 5,525)<br>OR (95% CI)	Model 2 (n = 5,525)<br>OR (95% CI)	Model 3 (n = 5,525)<br>OR (95% CI)	Model 4 (n = 5,280)<br>OR (95% CI)
Health literacy				
Adequate	Reference	Reference	Reference	Reference
Limited	1.493 (1.307 to 1.704)***	-	1.312 (1.143 to 1.506)***	1.179 (1.021 to 1.360)*
General cognitive ability†	-	0.741 (0.689 to 0.797)***	0.771 (0.715 to 0.832)***	0.843 (0.777 to 0.915)***
Age	0.951 (0.945 to 0.958)***	0.941 (0.933 to 0.948)***	0.941 (0.933 to 0.948)***	0.936 (0.927 to 0.945)***
Age^2	0.999 (0.999 to 1.000)**	0.999 (0.999 to 1.000)**	0.999 (0.999 to 1.000)***	0.999 (0.998 to 1.000)**
Sex				
Female	Reference	Reference	Reference	Reference
Male	0.744 (0.655 to 0.845)***	0.701 (0.617 to 0.797)***	0.707 (0.622 to 0.804)***	0.747 (0.654 to 0.854)***
Age left full-time education				
14 years or under	-	-	-	Reference
15-16 years	-	-	-	0.737 (0.596 to 0.912)**
17-18 years	-	-	-	0.522 (0.389 to 0.696)***

For peer review only - http://bmjopen.bmj.com/site/about/guidelines.xhtml

19 years or older	-	-	-	0.432 (0.315 to 0.591)***
Occupational class				
Managerial & professional	-	-	-	Reference
Intermediate	-	-	-	1.383 (1.138 to 1.680)**
Routine & manual	-	-	-	1.614 (1.352 to 1.930)***

*p<0.05, **p<0.01, ***p<0.001.

†ORs (95% CI) for general cognitive ability are the odds of reporting being a current smoker for a 1 SD increase in general cognitive ability.

For peer review only - http://bmjopen.bmj.com/site/about/guidelines.xhtml

Table 2 shows the odds ratios (ORs) and 95% confidence intervals (CIs) for reporting ever smoking. Adjusting for age and sex only, limited health literacy was associated with greater odds of ever smoking (model 1 OR=1.174, 95% CI 1.067 to 1.293). A 1SD higher score in general cognitive ability was associated with an 8.3% reduction in reporting ever smoking (model 2 OR=0.917, 95% CI 0.871 to 0.965). The associations between health literacy and general cognitive ability remain significant, though slightly reduced in size, in the model including both health literacy and cognitive ability (model 3). In model 4, which additionally adjusted for sociodemographic variables, the associations between health literacy and general cognitive ability with ever smoking were partly and fully attenuated, respectively, and no longer significant.

The ORs (95% CIs) for whether ever smokers reported being a current smoker at Wave 2 are shown in Table 3. For this analysis, a Box-Tidwell test revealed that models violated the assumption of linearity of the logit; therefore, an age-squared term was included in these models. To overcome multicollinearity, the ORs and CIs are based on models using centred continuous variables. Controlling for age and sex, having limited health literacy compared to adequate health literacy was associated with 49.3% greater odds of being a current smoker (model 1 OR=1.493, 95% CI 1.307 to 1.704). A 1SD higher score in general cognitive ability was associated with 25.9% lower odds of reporting being a current smoker (model 2 OR=0.741, 95% CI 0.689 to 0.797). Including both health literacy and general cognitive ability in model 3 reduced the size of the associations, but they remained significant. These associations continued to remain significant, though further attenuated, in the fully adjusted model, which additionally adjusted for age completed full-time education and occupational social class (model 4 OR for limited compared to adequate health literacy=1.179, 95% CI 1.021 to 1.360; OR for a 1SD higher score in general cognitive ability=0.843, 95% CI 0.777 to 0.915). In this final model, education and occupational social class were also significantly

For peer review only - http://bmjopen.bmj.com/site/about/guidelines.xhtml

associated with reporting being a current smoker. Compared to individuals who left full-time education at 14 years or under, those who left at age 17-18 or over 19 years had reduced odds of being a current smoker. Compared to those with a managerial or professional occupational class, those with a routine or manual occupational class had increased odds of being a current smoker.

DISCUSSION

This study found that health literacy and cognitive ability were independently related to smoking status in a sample of middle-aged and older adults residing in England. Participants with limited health literacy and lower cognitive ability were more likely to report having ever smoked. In ever smokers, those with limited health literacy and poorer cognitive ability were more likely to report that they continued to smoke. This latter association was independent of measures of socioeconomic status.

Whereas previous studies have found associations between health literacy and smoking,[13, 14] and cognitive ability and smoking,[3-10, 12] to the best of our knowledge, this is the first study to find that both health literacy and cognitive function are independently associated with reporting ever smoking and smoking cessation. Health literacy and cognitive function are strongly related[27-30] and some have proposed that health literacy is not a unique construct and is, rather, a subcomponent of general cognitive ability.[30] The current study, however, found that both health literacy and cognitive ability each play independent roles in predicting smoking which is in support of health literacy and cognitive ability being separate, albeit related, constructs.

A particularly important finding from the current study was that health literacy, independent of cognitive ability, education and occupational social class, was associated with whether

For peer review only - http://bmjopen.bmj.com/site/about/guidelines.xhtml

ever smokers continued to smoke. Health literacy, unlike already-established measures of socioeconomic status and perhaps more so than cognitive ability, is potentially modifiable.[31] Health literacy is thought to be a complex set of skills that include reading, numeracy, and health knowledge.[32] At least one component of health literacy—health knowledge—may be increased through educational programs and interventions[32] and this in turn can lead to improved health outcomes.[33] Future research should examine whether cognitive ability and health literacy play a role in the success of smoking interventions, and should investigate whether interventions designed to increase smoking-specific health knowledge increase smoking cessation in individuals with limited health literacy.

This cross-sectional study was interested in examining the characteristics of smokers and, although a relationship between cognitive ability, health literacy and smoking was identified, this study cannot determine the directionality of this association. It is possible that smokers have lower cognitive ability and health literacy because smoking has damaging effects on both health literacy and cognitive ability. It is equally possible that individuals who are less intelligent and less health literate are more likely to start smoking in the first place, and less likely to quit. For cognitive ability, evidence suggests that both of these pathways may be at least partially correct. Individuals with higher cognitive ability early in life are less likely to start smoking and more likely to quit,[10, 12] and smoking may cause steeper cognitive change throughout life.[4-7] A similar relationship may exist between health literacy and smoking. Further longitudinal studies which include measures of cognitive ability and health literacy in early life are needed to understand the pathways between health literacy, cognitive ability and smoking.

The key strengths of this study include the large sample size and the fact that ELSA was designed to be representative of individuals aged over 50 residing in England.[19] One limitation is that smoking status is self-reported; however, self-reported smoking has been

For peer review only - http://bmjopen.bmj.com/site/about/guidelines.xhtml

found to be in agreement with serum cotinine measurements.[34] The smoking measures used here do not take into account the amount smoked throughout life. These results reported here may underestimate the true effect sizes because lifetime smoking was not considered. Another limitation is that the cognitive ability and health literacy tests were brief. Health literacy was assessed using a four-item test, and the measure of general cognitive ability created here did not include tests of reasoning and knowledge—measures which load highly on general cognitive ability.[35] Given other studies which have suggested that some health literacy measures are essentially aspects of cognitive function,[30, 36] and given also the limited cognitive test battery used in ELSA, it is possible that some of the independent contribution of the health literacy measure here is residual cognitive capability not picked up by the general cognitive ability component.

In this study of middle-aged and older adults, lower cognitive ability and poorer health literacy were associated with reporting ever smoking, and with whether ever smokers continued to smoke. The latter association was independent of socioeconomic variables. Further research is needed to identify possible pathways between health literacy, cognitive function and starting and quitting smoking.

For peer review only

For peer review only - http://bmjopen.bmj.com/site/about/guidelines.xhtml

Acknowledgements We are grateful to the ELSA participants, and to the UK Data Archive for supplying the data.

Contributors CFR discussed and planned the study and analyses, analysed the data, interpreted the data, and drafted the initial manuscript. JMS discussed and planned the study and analysis, interpreted the data, and contributed to the manuscript. IJD discussed and planned the study and analysis, interpreted the data, and contributed to the manuscript.

Funding This work was supported by the University of Edinburgh Centre for Cognitive Ageing and Cognitive Epidemiology, part of the cross council Lifelong Health and Wellbeing Initiative, funded by the Biotechnology and Biological Sciences Research Council (BBSRC), and Medical Research Council (MRC) (grant number MR/K026992/1).

Ethical approval Ethical approval for the English Longitudinal Study of Ageing was obtained from the National Research and Ethics Committee.

Competing interests None declared.

Data sharing statement No additional data available. Anonymised data from the English Longitudinal Study of Ageing is available from the UK Data Service (https://www.ukdataservice.ac.uk/).

For peer review only

For peer review only - http://bmjopen.bmj.com/site/about/guidelines.xhtml

REFERENCES

1 Office for National Statistics. Adult smoking habits in the UK: 2016. Retrieved from: https://www.ons.gov.uk/peoplepopulationandcommunity/healthandsocialcare/healthandlifeexpectancies/bulletins/adultsmokinghabitsingreatbritain/2016. England: Public Health England 2017 (accessed January 2018).

2 Allender S, Balakrishnan R, Scarborough P, et al. The burden of smoking-related ill health in the UK. *Tob Control* 2009;18(4):262-7. doi:10.1136/tc.2008.026294 [published Online First: 9 June 2009].

3 Mons U, Schottker B, Muller H, et al. History of lifetime smoking, smoking cessation and cognitive function in the elderly population. *Eur J Epidemiol* 2013;28(10):823-31. doi:10.1007/s10654-013-9840-9 [published Online First: 30 August 2013].

4 Reitz C, Luchsinger J, Tang MX, et al. Effect of smoking and time on cognitive function in the elderly without dementia. *Neurology* 2005;65(6):870-5. doi:10.1212/01.wnl.0000176057.22827.b7 [published Online First: 26 September 2005].

5 Richards M, Jarvis MJ, Thompson N, et al. Cigarette smoking and cognitive decline in midlife: evidence from a prospective birth cohort study. *Am J Public Health* 2003;93(6):994-8. doi:10.2105/AJPH.93.6.994.

6 Anstey KJ, von Sanden C, Salim A, et al. Smoking as a risk factor for dementia and cognitive decline: a meta-analysis of prospective studies. *Am J Epidemiol* 2007;166(4):367-78. doi:10.1093/aje/kwm116[published Online First: 14 June 2007].

7 Deary IJ, Pattie A, Taylor MD, et al. Smoking and cognitive change from age 11 to age 80. *J Neurol Neurosurg Psychiatry* 2003;74(7):1006-7. doi:10.1136/jnnp.74.7.1006.

8 Peters R, Poulter R, Warner J, et al. Smoking, dementia and cognitive decline in the elderly, a systematic review. *BMC Geriatr* 2008;8:36. doi:10.1186/1471-2318-8-36.

For peer review only - http://bmjopen.bmj.com/site/about/guidelines.xhtml

9 Corley J, Gow AJ, Starr JM, et al. Smoking, childhood IQ, and cognitive function in old age. *J Psychosom Res* 2012;73(2):132-8. doi:10.1016/j.jpsychores.2012.03.006 [published Online First: 26 April 2012].

10 Batty GD, Deary IJ, Schoon I, et al. Mental ability across childhood in relation to risk factors for premature mortality in adult life: the 1970 British Cohort Study. *J Epidemiol Community Health* 2007;61(11):997-1003. doi:10.1136/jech.2006.054494.

11 Hemmingsson T, Kriebel D, Melin B, et al. How does IQ affect onset of smoking and cessation of smoking-linking the Swedish 1969 conscription cohort to the Swedish survey of living conditions. *Psychosom Med* 2008;70(7):805-10. doi:10.1097/PSY.0b013e31817b955f [published Online First: 7 July 2008].

12 Taylor MD, Hart CL, Davey Smith G, et al. Childhood mental ability and smoking cessation in adulthood: prospective observational study linking the Scottish Mental Survey 1932 and the Midspan studies. *J Epidemiol Community Health* 2003;57(6):464-5. doi:10.1136/jech.57.6.464.

13 Fernandez DM, Larson JL, Zikmund-Fisher BJ. Associations between health literacy and preventive health behaviors among older adults: findings from the health and retirement study. *BMC Public Health* 2016;16:596. doi:10.1186/s12889-016-3267-7.

14 von Wagner C, Knight K, Steptoe A, et al. Functional health literacy and health-promoting behaviour in a national sample of British adults. *J Epidemiol Community Health* 2007;61(12):1086-90. doi:10.1136/jech.2006.053967.

15 Geboers B, Reijneveld SA, Jansen CJ, et al. Health Literacy Is Associated With Health Behaviors and Social Factors Among Older Adults: Results from the LifeLines Cohort Study. *J Health Commun* 2016;21(sup2):45-53. doi:10.1080/10810730.2016.1201174 [published Online First: 23 September 2016].

For peer review only - http://bmjopen.bmj.com/site/about/guidelines.xhtml

16 Wolf MS, Gazmararian JA, Baker DW. Health literacy and health risk behaviors among older adults. *Am J Prev Med* 2007;32(1):19-24. doi:10.1016/j.amepre.2006.08.024 [published Online First: 20 Dec 2006].

17 Institute of Medicine. Health Literacy: A Prescription to End Confusion. Washington DC: National Academies Press 2004.

18 Baker DW. The meaning and the measure of health literacy. *J Gen Intern Med* 2006;21(8):878-83. doi:10.1111/j.1525-1497.2006.00540.x.

19 Steptoe A, Breeze E, Banks J, et al. Cohort profile: the English longitudinal study of ageing. *Int J Epidemiol* 2013;42(6):1640-8. doi:10.1093/ije/dys168 [published Online First: 9 November 2012].

20 Thorn W. International Adult Literacy and Basic Skills Surveys in the OECD Regresion. OECD Educatio Working Papers. Paris: OECD Publishing 2009.

21 Bostock S, Steptoe A. Association between low functional health literacy and mortality in older adults: longitudinal cohort study. *BMJ* 2012;344:e1602. doi:10.1136/bmj.e1602.

22 Kobayashi LC, Wardle J, von Wagner C. Limited health literacy is a barrier to colorectal cancer screening in England: evidence from the English Longitudinal Study of Ageing. *Prev Med* 2014;61:100-5. doi:10.1016/j.ypmed.2013.11.012 [published Online First: 25 November 2013].

23 Gale CR, Deary IJ, Wardle J, et al. Cognitive ability and personality as predictors of participation in a national colorectal cancer screening programme: the English Longitudinal Study of Ageing. *J Epidemiol Community Health* 2015;69(6):530. doi:10.1136/jech-2014-204888 [published Online First: 3 February 2015].

24 Salthouse TA. Selective review of cognitive aging. *J Int Neuropsychol Soc* 2010;16(5):754-60. doi:10.1017/s1355617710000706 [published Online First: 2 August 2010].

For peer review only

For peer review only - http://bmjopen.bmj.com/site/about/guidelines.xhtml

25 Park DC, Reuter-Lorenz P. The adaptive brain: aging and neurocognitive scaffolding. *Annu Rev Psychol* 2009;60:173-96. doi:10.1146/annurev.psych.59.103006.093656.

26 Rose D, Pevalin DJ, O'Reilly K. The National Statistics Socio-economic Classification: origins, development and use. Basingstoke, UK: Palgrave Macmillan 2005.

27 Federman AD, Sano M, Wolf MS, et al. Health literacy and cognitive performance in older adults. *J Am Geriatr Soc* 2009;57(8):1475-80. doi:10.1111/j.1532-5415.2009.02347.x [published Online First: 8 June 2009].

28 Kobayashi LC, Wardle J, Wolf MS, et al. Cognitive Function and Health Literacy Decline in a Cohort of Aging English Adults. *J Gen Intern Med* 2015;30(7):958-64. doi:10.1007/s11606-015-3206-9 [published Online First: 14 February 2015].

29 Murray C, Johnson W, Wolf MS, et al. The Association between Cognitive Ability across the Lifespan and Health Literacy in Old Age: The Lothian Birth Cohort 1936. *Intelligence* 2011;39(4):178-87. doi:10.1016/j.intell.2011.04.001 [published Online First: 4 May 2011].

30 Reeve CL, Basalik D. Is health literacy an example of construct proliferation? A conceptual and empirical evaluation of its redundancy with general cognitive ability. *Intelligence* 2014;44:93-102. doi:10.1016/j.intell.2014.03.004 [published Online First: 16 April 2014].

31 Nutbeam D. The evolving concept of health literacy. *Soc Sci Med* 2008;67(12):2072-8. doi:10.1016/j.socscimed.2008.09.050 [published Online First: 25 October 2008].

32 Chin J, Morrow DG, Stine-Morrow EA, et al. The process-knowledge model of health literacy: evidence from a componential analysis of two commonly used measures. *J Health Commun* 2011;16 Suppl 3:222-41. doi:10.1080/10810730.2011.604702.

33 Rothman RL, DeWalt DA, Malone R, et al. Influence of patient literacy on the effectiveness of a primary care-based diabetes disease management program. *JAMA* 2004;292(14):1711-6. doi:10.1001/jama.292.14.1711.

For peer review only - http://bmjopen.bmj.com/site/about/guidelines.xhtml

For peer review only

34 Vartiainen E, Seppala T, Lillsunde P, et al. Validation of self reported smoking by serum cotinine measurement in a community-based study. *J Epidemiol Community Health* 2002;56(3):167-70. doi:10.1136/jech.56.3.167.

35 Salthouse TA. Localizing age-related individual differences in a hierarchical structure. *Intelligence* 2004;32(6). doi:10.1016/j.intell.2004.07.003 [published Online First: 11 September 2004].

36 Mottus R, Johnson W, Murray C, et al. Towards understanding the links between health literacy and physical health. *Health Psychology* 2014;33(2):164-73. doi:10.1037/a0031439.

For peer review only

STROBE 2007 (v4) Statement—Checklist of items that should be included in reports of *cohort studies*

Section/Topic	Item #	Recommendation	Reported on page #
**Title and abstract**	1	(*a*) Indicate the study's design with a commonly used term in the title or the abstract	1
		(*b*) Provide in the abstract an informative and balanced summary of what was done and what was found	2
**Introduction**			
Background/rationale	2	Explain the scientific background and rationale for the investigation being reported	3-4
Objectives	3	State specific objectives, including any prespecified hypotheses	4
**Methods**			
Study design	4	Present key elements of study design early in the paper	4-7
Setting	5	Describe the setting, locations, and relevant dates, including periods of recruitment, exposure, follow-up, and data collection	4-5
Participants	6	(*a*) Give the eligibility criteria, and the sources and methods of selection of participants. Describe methods of follow-up	4-5
		(*b*) For matched studies, give matching criteria and number of exposed and unexposed	NA
Variables	7	Clearly define all outcomes, exposures, predictors, potential confounders, and effect modifiers. Give diagnostic criteria, if applicable	5-7
Data sources/ measurement	8*	For each variable of interest, give sources of data and details of methods of assessment (measurement). Describe comparability of assessment methods if there is more than one group	4-7
Bias	9	Describe any efforts to address potential sources of bias	NA
Study size	10	Explain how the study size was arrived at	4-5, 8
Quantitative variables	11	Explain how quantitative variables were handled in the analyses. If applicable, describe which groupings were chosen and why	7
Statistical methods	12	(*a*) Describe all statistical methods, including those used to control for confounding	7
		(*b*) Describe any methods used to examine subgroups and interactions	7
		(*c*) Explain how missing data were addressed	8
		(*d*) If applicable, explain how loss to follow-up was addressed	NA
		(*e*) Describe any sensitivity analyses	NA
**Results**			

For peer review only

Participants	13*	(a) Report numbers of individuals at each stage of study—eg numbers potentially eligible, examined for eligibility, confirmed eligible, included in the study, completing follow-up, and analysed	4-5, 8
		(b) Give reasons for non-participation at each stage	4-5, 8
		(c) Consider use of a flow diagram	NA
Descriptive data	14*	(a) Give characteristics of study participants (eg demographic, clinical, social) and information on exposures and potential confounders	8, 10-11
		(b) Indicate number of participants with missing data for each variable of interest	10-11
		(c) Summarise follow-up time (eg, average and total amount)	NA
Outcome data	15*	Report numbers of outcome events or summary measures over time	8, 10-11
Main results	16	(*a*) Give unadjusted estimates and, if applicable, confounder-adjusted estimates and their precision (eg, 95% confidence interval). Make clear which confounders were adjusted for and why they were included	12-15
		(*b*) Report category boundaries when continuous variables were categorized	NA
		(*c*) If relevant, consider translating estimates of relative risk into absolute risk for a meaningful time period	NA
Other analyses	17	Report other analyses done—eg analyses of subgroups and interactions, and sensitivity analyses	NA
**Discussion**			
Key results	18	Summarise key results with reference to study objectives	16
**Limitations**			
Interpretation	20	Give a cautious overall interpretation of results considering objectives, limitations, multiplicity of analyses, results from similar studies, and other relevant evidence	16-18
Generalisability	21	Discuss the generalisability (external validity) of the study results	17-18
**Other information**			
Funding	22	Give the source of funding and the role of the funders for the present study and, if applicable, for the original study on which the present article is based	19

*Give information separately for cases and controls in case-control studies and, if applicable, for exposed and unexposed groups in cohort and cross-sectional studies.

Note: An Explanation and Elaboration article discusses each checklist item and gives methodological background and published examples of transparent reporting. The STROBE checklist is best used in conjunction with this article (freely available on the Web sites of PLoS Medicine at http://www.plosmedicine.org/, Annals of Internal Medicine at http://www.annals.org/, and Epidemiology at http://www.epidem.com/). Information on the STROBE Initiative is available at www.strobe-statement.org.

For peer review only

BMJ Open

The role of health literacy and cognitive ability in smoking: a cross-sectional analysis of the English Longitudinal Study of Ageing

Journal:	*BMJ Open*
Manuscript ID	bmjopen-2018-023929.R1
Article Type:	Research
Date Submitted by the Author:	25-Jul-2018

Complete List of Authors:	Fawns-Ritchie, Chloe; University of Edinburgh, Centre for Cognitive Ageing and Cognitive Epidemiology<br>Starr, John; University of Edinburgh<br>Deary, Ian; University of Edinburgh, Centre for Cognitive Ageing and Cognitive Epidemiology, Department of Psychology

**Primary Subject Heading**:	Epidemiology
Secondary Subject Heading:	Smoking and tobacco
Keywords:	EPIDEMIOLOGY, STATISTICS & RESEARCH METHODS, PUBLIC HEALTH

SCHOLARONE™ Manuscripts

For peer review only

The role of health literacy and cognitive ability in smoking: a cross-sectional analysis of the English Longitudinal Study of Ageing

Chloe Fawns-Ritchie[1,2], John M Starr[1,3], and Ian J Deary[1,2*]

[1]Centre for Cognitive Ageing and Cognitive Epidemiology, University of Edinburgh, Edinburgh, UK

[2]Department of Psychology, University of Edinburgh, Edinburgh, UK

[3]Alzheimer Scotland Dementia Research Centre, University of Edinburgh, Edinburgh, UK

*Corresponding author: Ian J Deary, Centre for Cognitive Ageing and Cognitive Epidemiology, Department of Psychology, University of Edinburgh

7 George Square

Edinburgh, EH8 9JZ

Scotland, UK

Telephone: +44 131 650 3452

ian.deary@ed.ac.uk

Word count: 3,429

For peer review only

ABSTRACT

Objectives We used logistic regression to investigate whether health literacy and cognitive ability independently predicted whether participants have ever smoked and, in ever smokers, whether participants still smoked nowadays.

Design Cross-sectional study.

Setting This study used data from Wave 2 (2004-05) of the English Longitudinal Study of Ageing, which is a cohort study of adults who live in England and who, at baseline, were aged 50 years and older.

Participants 8,734 (mean age = 65.31 years, SD = 10.18) English Longitudinal Study of Ageing participants who answered questions about their current and past smoking status, and completed cognitive ability and health literacy tests at Wave 2.

Primary and secondary outcome measures The primary outcome measures were whether participants reported ever smoking at Wave 2 and whether ever smokers reported still smoking at Wave 2.

Results In models adjusting for age, sex, age left full-time education, and occupational social class, limited health literacy (OR = 1.096, 95% CI 0.988 to 1.216) and higher general cognitive ability (OR = 1.000, 95% CI 0.945 to 1.057) were not associated with reporting ever smoking. In ever smokers, limited compared to adequate health literacy was associated with greater odds of being a current smoker (OR = 1.194, 95% CI 1.034 to 1.378) and a 1SD higher general cognitive ability score was associated with reduced odds of being a current smoker (OR = 0.878, 95% CI 0.810 to 0.951), when adjusting for age, sex, age left full-time education, and occupational social class.

For peer review only - http://bmjopen.bmj.com/site/about/guidelines.xhtml

Conclusions When adjusting for education and occupation variables, this study found that health literacy and cognitive ability were independently associated with whether ever smokers continued to smoke nowadays, but not with whether participants had ever smoked.

For peer review only

For peer review only - http://bmjopen.bmj.com/site/about/guidelines.xhtml

Strengths and limitations of this study

- This study used a large sample (n = 8,734) from the English Longitudinal Study of Ageing; a study designed to be representative of the English population aged over 50 years.
- This analysis was cross-sectional and therefore cannot determine the direction of the association between smoking, health literacy and cognitive ability.
- This study included measures of both health literacy and cognitive ability which allowed us to investigate whether health literacy was associated with smoking status when controlling for cognitive ability.
- Smoking status was self-reported.

For peer review only

For peer review only - http://bmjopen.bmj.com/site/about/guidelines.xhtml

INTRODUCTION

The effects of smoking on ill-health have been known for decades. The prevalence of smoking in the UK is falling and the number of smokers who are quitting is increasing.[1] Despite this, nearly 16% of the UK population were current smokers in 2016[1] and smoking remains one the largest causes of preventable morbidity and mortality in the UK.[1, 2] Understanding the characteristics of individuals who take up smoking, and who quit smoking is important to be able to design and target smoking education and interventions.

Cognitive ability is associated with smoking. Individuals who smoke have lower scores on cognitive tests than those who have never smoked.[3-5] Smokers show steeper ageing-related cognitive decline[4-7] and have increased risk of Alzheimer's disease,[6, 8] compared to non-smokers. One possible pathway between smoking and cognitive ability is that smoking has harmful consequences for the vascular system, which could in turn affect cognitive functioning.[6, 9]

A perhaps complementary explanation is that individuals who have lower cognitive ability in youth are more likely to take up smoking and less likely to quit.[9-11] Corley et al.[9] found that, when controlling for childhood cognitive ability, the association between smoking and cognitive function in old age was attenuated, and in some cases, became non-significant. Two studies[10, 12] found different patterns when investigating the relationship between childhood cognitive ability and reporting ever smoking. One study using the 1970 British Birth Cohort[10] found that individuals with higher childhood cognitive ability were less likely to have ever smoked in a sample of middle-aged participants, whereas another report, based on two of the Midspan prospective cohort studies[12], found no association between cognitive ability in childhood and ever smoking in a sample of older adults. Both these

For peer review only - http://bmjopen.bmj.com/site/about/guidelines.xhtml

studies, however, found that among ever smokers, individuals with higher childhood cognitive ability were more likely to quit smoking.[10, 12]

A person's health literacy may also play a role in smoking status, though the evidence for an association between health literacy and smoking is mixed.[13-16] Health literacy is the capacity to acquire, process and use health information to successfully navigate all aspects of health, including the ability to use health documents, interact with health care professionals, and undertake health promoting behaviours to prevent future ill-health.[17, 18] Some studies have found that individuals with lower health literacy are more likely to smoke,[13, 14] whereas others have not.[15, 16] It is possible that individuals who have limited health literacy are less aware of the adverse effects of smoking on health, and may be less able to understand and utilise smoking cessation services.

The current study sought to determine whether health literacy and cognitive ability, when studied together, have independent associations with smoking. Drawing on the English Longitudinal Study of Ageing (ELSA), we first investigated whether health literacy and cognitive ability were independently associated with whether individuals had ever smoked. Second, we investigated whether there was a relationship between health literacy, cognitive ability and whether ever smokers continued to smoke, or quit.

METHODS

Participants

This study used data from ELSA, a panel study designed to be representative of individuals aged 50 years and older living in England.[19] A total of 11,391 participants took part in Wave 1 in 2002-03. Wave 1 participants were individuals who had previously taken part in the Health Survey for England, were born before 1 March 1952, and were living in a private

For peer review only - http://bmjopen.bmj.com/site/about/guidelines.xhtml

household in England at the first wave.[19] These participants have been followed up every two years, and the sample has been refreshed at subsequent waves to maintain a representative sample of participants aged over 50 years. More information on this cohort is provided elsewhere.[19] The current sample consists of participants who completed the Wave 2 (2004-05) interview (n = 8,780); this is the first wave in which health literacy was assessed. Ethical approval was granted from the National Research and Ethics Committee. This study conformed to the principles embodied in the Declaration of Helsinki.

For peer review only

Measures

ELSA interviews were carried out using computer-assisted interviewing in the participants own home.

Smoking

Two aspects of smoking status (ever versus never smoker and current versus former smoker) were the outcome variables in these analyses. Participants were asked 'Have you ever smoked cigarettes?'. Participants were categorised as ever smokers if they answered 'yes' and never smokers if they answered 'no' at either Wave 1 or 2. Ever smokers were additionally asked 'Do you smoke cigarettes at all nowadays?'. Ever smokers who answered 'yes' to smoking cigarettes nowadays at Wave 2 were categorised as current smokers, whereas ever smokers who answered 'no' were categorised as former smokers.

Health literacy

Health literacy was assessed at Wave 2 using a four-item comprehension test previously used in the International Adult Literacy Survey.[20] Participants were presented with a piece of paper containing instructions similar to those that would be found on a packet of over-the-

For peer review only - http://bmjopen.bmj.com/site/about/guidelines.xhtml

counter medication. Participants were instructed to read the medicine label and were then asked four questions about the information on this label (e.g., ‘what is the maximum number of days you may take this medicine?’). The label was available to the participant to refer to at any time. This task was designed to measure the skills thought to be required to understand and use health materials correctly, such as the ability to read and use numbers in a health context.[21] One point was awarded for each correctly answered question (range 0-4). As has been done in previous ELSA studies[22, 23] health literacy scores were categorised as ‘adequate’ (4/4 correct) or ‘limited’ (≤ 3 correct).

Cognitive function

Four tests of cognitive function that were administered at Wave 2 of the ELSA study were used here. These tests are thought to assess episodic memory, executive function and processing speed; these are cognitive domains which tend to decline on average with increasing age.[24, 25] In the word list recall test, participants heard a list of 10 words which they had to recall immediately (immediate recall test) and again after a short delay (delayed recall test). The score on each occasion was the number of words remembered (range 0-10). Executive function was assessed using categorical verbal fluency (number of animals named in 60 seconds). The letter cancellation test, in which participants were to scan rows of letters and score out all P’s and W’s, was used to measure processing speed. The score is the number of P’s and W’s scored out in 60 seconds. Exploratory factor analysis (EFA) using principal axis factoring was used to derive a composite measure of general cognitive ability. Scores on the four cognitive tests were entered into the EFA. Prior to this, individuals who scored 0 or greater than 4 SD above the mean on the animal fluency test and the letter cancellation test were removed. Scores of 0 indicate that the participant did not understand the task, and scores 4 SD above the mean were seen as dubiously high given the one minute time limit for these tests. One unrotated factor was extracted which accounted for 44% of the

For peer review only - http://bmjopen.bmj.com/site/about/guidelines.xhtml

total variance in the four cognitive tests. The loadings of the tests were: immediate word recall = 0.78; delayed word recall = 0.83; animal naming = 0.53; letter cancellation = 0.42. This factor score was converted to a z-score (mean = 0.00, SD = 1.00) and was used as a measure of general cognitive ability.

Covariates

Age in years, sex, age of leaving full-time education and occupational social class were used as covariates. For confidentially reasons, owing to there being few of them, participants aged over 90 years have had their age set to 90. Participants were asked at what age they left continuous full-time education (recorded as not yet finished, never went to school, 14 or under, at 15, at 16, at 17, at 18, and 19 or over). For the purpose of this study, age of leaving full-time education was categorised as 14 years or under, 15-16 years, 17-18 years, and 19 years or over. Occupational social class was categorised using the National Statistics Socio-economic classification (NS-SEC) 3-cateogries: managerial and professional, intermediate, and routine and manual.[26]

Patient and public involvement

Participants were not involved in the development of any part of this study.

Statistical analysis

Two sets of analyses were carried out. First, ever smokers were compared with never smokers; second, current smokers were compared with former smokers. To determine whether ever versus never smokers, and current versus former smokers differ on health literacy, general cognitive ability, and sociodemographic variables, t-tests were used for

For peer review only - http://bmjopen.bmj.com/site/about/guidelines.xhtml

normally-distributed continuous variables, Mann-Whitney U tests were used for non-normal continuous variables, and χ^2 tests were used for categorical variables. Rank order correlations were calculated between the predictor variables to examine any bivariate associations between these variables. Binary logistic regression was used to examine the independent associations of health literacy and general cognitive ability on smoking status. Age and sex were entered in all models. Health literacy and general cognitive ability were entered individually in models 1 and 2, respectively. To determine whether both health literacy and general cognitive ability are independently associated with smoking, both predictors were included in model 3. Model 4 additionally adjusted for age of finishing full-time education and occupational social class to determine whether any associations between health literacy, cognitive function and smoking remained after controlling for these sociodemographic variables.

RESULTS

Of the 8,780 participants who completed the Wave 2 interview, 8,734 participants had complete data on smoking, cognitive ability, and health literacy, and they make up the analytic sample. Participant characteristics are reported in Table 1. A total of 5,525 (63.3%) participants reported ever smoking, whereas 3,209 (36.7%) participants reported having never smoked. Ever smokers were more likely to have limited health literacy and have a lower general cognitive ability than never smokers. Ever smokers were older, were more likely to be male, have left full-time education at a younger age, and have a lower occupational social class than never smokers. A total of 1,356 (15.5%) participants reported that they still smoked cigarettes at Wave 2, whereas 4,169 (47.7%) participants reported that they had stopped. Current smokers were more likely to have limited health literacy than

For peer review only - http://bmjopen.bmj.com/site/about/guidelines.xhtml

former smokers; however, the two groups did not differ on general cognitive ability. Current smokers were younger, more likely to be female, have left full-time education at a younger age, and to have a lower occupational social class than former smokers. Given that current smokers were, on average, 4.5 years younger than former smokers, we tested the point-biseral correlation between smoking status and general cognitive ability, with and without controlling for age. When not controlling for age, the correlation between smoking and general cognitive ability was 0.01 ($p = 0.389$). Adjusting for age, the correlation was -0.09 and this was significant ($p < 0.001$).

Rank order correlations between the predictor variables are shown in Table 2. All predictor variables were significantly correlated with each other, with the exception of sex with health literacy and education. Having adequate health literacy was moderately associated with having higher general cognitive ability ($r = 0.31$, $p < 0.001$). Adequate health literacy was associated with having higher qualifications ($r = 0.23$, $p < 0.001$) and a higher occupational class ($r = -0.18$, $p < 0.001$). Older adults were less likely to have adequate health literacy ($r = 0.16$, $p < 0.001$). General cognitive ability was strongly correlated with age. Older individuals tended to have lower general cognitive ability ($r = -0.46$, $p < 0.001$). Female participants ($r = -0.10$, $p < 0.001$), individuals with higher qualifications ($r = 0.38$, $p < 0.001$), and higher occupational class ($r = -0.25$, $p < 0.001$) tended to have higher general cognitive ability.

For peer review only

Table 1: Participant characteristics according to smoking status (n = 8,734)*

	Smoking history			Smoking cessation†		
	Ever smoker (n = 5,525)	Never smoker (n = 3,209)	P-value for difference	Current smoker (n = 1,356)	Former smoker (n = 4,169)	P value for difference
Health literacy, n (%)			0.001			< 0.001
Adequate	3,647 (66.0)	2,233 (69.6)		840 (61.9)	2,807 (67.3)	
Limited	1,878 (34.0)	976 (30.4)		516 (38.1)	1,362 (32.7)	
General cognitive ability, mean (SD)	-0.04 (1.00)	0.08 (0.99)	< 0.001	-0.02 (0.99)	-0.05 (1.01)	0.385
Age (years), mean (SD)	65.53 (10.13)	64.93 (10.24)	0.005	62.12 (9.12)	66.64 (10.20)	< 0.001
Sex, n (%)			0.001			< 0.001
Female	2,752 (49.8)	2,172 (67.7)		761 (56.1)	1,991 (47.8)	
Male	2,773 (50.2)	1,037 (32.3)		595 (43.9)	2,178 (52.2)	
Age left full-time education, n (%)			< 0.001			< 0.001
14 years or under	1,104 (20.6)	553 (17.6)		233 (17.7)	871 (21.5)	
15-16 years	2,936 (54.8)	1,578 (50.2)		856 (65.0)	2,080 (51.4)	
17-18 years	665 (12.4)	488 (15.5)		128 (9.7)	537 (13.3)	

For peer review only - http://bmjopen.bmj.com/site/about/guidelines.xhtml

19 years or over	657 (12.3)	526 (16.7)		99 (7.5)	558 (13.8)	
Occupational social class, n (%)			< 0.001			< 0.001
Managerial & professional	1,677 (30.8)	1,047 (33.2)		274 (20.6)	1,403 (34.2)	
Intermediate	1,263 (23.2)	884 (28.1)		312 (23.4)	951 (23.2)	
Routine & manual	2,499 (45.9)	1,218 (38.7)		747 (56.0)	1,752 (42.7)	

*Characteristics for age left full-time education are based on a subset of 8,507 participants with this data and characteristics for occupational social class are based on a subset of 8,588 participants with this data.

†For smoking cessation comparisons, the ever smoker category is divided into whether ever smokers are current or former smokers.

For peer review only

Table 2: Rank order correlations between predictor variables (pairwise n = 8367-8734)

	Health literacy	General cognitive ability	Age (years)	Sex	Education	Occupational class
Health literacy	-					
General cognitive ability	0.31***	-				
Age (years)	-0.16***	-0.46***	-			
Sex	0.01	-0.10***	0.02*	-		
Education	0.23***	0.38***	-0.40***	0.00	-	
Occupational class	-0.18***	-0.25***	0.07***	-0.09***	-0.41***	-

*p<0.05, **p<0.01, *** p<0.001.

Health literacy was coded 0 for inadequate health literacy, 1 for adequate health literacy; sex was coded 0 for female, 1 for male; education is age left full-time education and was coded 1 for 14 years or under, 2 for 15-16 years, 3 for 17-18 years, 4 for 19 years or older; occupational social class was coded 1 for managerial and professional, 2 for intermediate, 3 for routine and manual.

For peer review only - http://bmjopen.bmj.com/site/about/guidelines.xhtml

Table 3: Odds ratios and 95% CIs from logistic regression models of whether participants have ever smoked

	Model 1 (n = 8,734)<br>OR (95% CI)	Model 2 (n = 8,734)<br>OR (95% CI)	Model 3 (n = 8,734)<br>OR (95% CI)	Model 4 (n = 8,367)<br>OR (95% CI)
Health literacy				
Adequate	Reference	Reference	Reference	Reference
Limited	1.174 (1.067 to 1.293)**	-	1.134 (1.026 to 1.254)*	1.096 (0.988 to 1.216)
General cognitive ability†	-	0.919 (0.874 to 0.967)**	0.936 (0.888 to 0.987)*	1.000 (0.945 to 1.057)
Age (years)	1.004 (1.000 to 1.008)	1.001 (0.996 to 1.006)	1.001 (0.996 to 1.006)	1.002 (0.996 to 1.007)
Sex				
Female	Reference	Reference	Reference	Reference
Male	2.112 (1.929 to 2.313)***	2.077 (1.896 to 2.276)***	2.087 (1.905 to 2.288)***	2.150 (1.955 to 2.366)***
Age left full-time education				
14 years or under	-	-	-	Reference
15-16 years	-	-	-	1.016 (0.880 to 1.172)
17-18 years	-	-	-	0.828 (0.690 to 0.994)*
19 years or older	-	-	-	0.693 (0.572 to 0.839)***

For peer review only - http://bmjopen.bmj.com/site/about/guidelines.xhtml

Occupational class				
Managerial & professional	-	-	-	Reference
Intermediate	-	-	-	0.919 (0.810 to 1.041)
Routine & manual	-	-	-	1.204 (1.066 to 1.360)**

*p<0.05, **p<0.01, *** p<0.001.

†ORs (95% CIs) for general cognitive ability are the odds of reporting ever smoking for a 1 SD increase in general cognitive ability.

For peer review only - http://bmjopen.bmj.com/site/about/guidelines.xhtml

Table 4: Odds ratios and 95% CIs from logistic regression models of whether ever smokers still smoke nowadays

	Model 1 (n = 5,525)<br>OR (95% CI)	Model 2 (n = 5,525)<br>OR (95% CI)	Model 3 (n = 5,525)<br>OR (95% CI)	Model 4 (n = 5,280)<br>OR (95% CI)
Health literacy				
Adequate	Reference	Reference	Reference	Reference
Limited	1.493 (1.307 to 1.704)***	-	1.338 (1.165 to 1.536)***	1.194 (1.034 to 1.378)*
General cognitive ability†	-	0.772 (0.718 to 0.829)***	0.805 (0.747 to 0.868)***	0.878 (0.810 to 0.951)**
Age	0.952 (0.945 to 0.958)***	0.943 (0.936 to 0.951)***	0.943 (0.936 to 0.950)***	0.938 (0.929 to 0.947)***
Age^2	0.999 (0.999 to 1.000)**	0.999 (0.999 to 1.000)**	0.999 (0.999 to 1.000)**	0.999 (0.998 to 1.000)**
Sex				
Female	Reference	Reference	Reference	Reference
Male	0.744 (0.655 to 0.845)***	0.707 (0.622 to 0.803)***	0.714 (0.628 to 0.811)***	0.755 (0.661 to 0.863)***
Age left full-time education				
14 years or under	-	-	-	Reference
15-16 years	-	-	-	0.734 (0.593 to 0.908)**
17-18 years	-	-	-	0.515 (0.384 to 0.687)***

For peer review only - http://bmjopen.bmj.com/site/about/guidelines.xhtml

19 years or older	-	-	-	0.432 (0.308 to 0.578)***
Occupational class				
Managerial & professional	-	-	-	Reference
Intermediate	-	-	-	1.390 (1.144 to 1.689)***
Routine & manual	-	-	-	1.614 (1.375 to 1.961)***

*p<0.05, **p<0.01, ***p<0.001.

†ORs (95% CI) for general cognitive ability are the odds of reporting being a current smoker for a 1 SD increase in general cognitive ability.

For peer review only - http://bmjopen.bmj.com/site/about/guidelines.xhtml

For peer review only

Table 3 shows the odds ratios (ORs) and 95% confidence intervals (CIs) for reporting ever smoking. Adjusting for age and sex only, limited health literacy was associated with greater odds of ever smoking (model 1 OR = 1.174, 95% CI 1.067 to 1.293). A 1SD higher score in general cognitive ability was associated with an 8.1% reduction in reporting ever smoking (model 2 OR = 0.919, 95% CI 0.874 to 0.967). The associations between health literacy and general cognitive ability with ever smoking remain significant, though slightly reduced in size, in the model including both health literacy and cognitive ability (model 3). In model 4, which additionally adjusted for sociodemographic variables, the associations between health literacy (OR = 1.096, 95% CI 0.988 to 1.216) and general cognitive ability (OR = 1.000, 95% CI 0.945 to 1.057) with ever smoking were partly and fully attenuated, respectively, and no longer significant.

The ORs (95% CIs) for whether ever smokers reported being a current smoker at Wave 2 are shown in Table 4. For this analysis, a Box-Tidwell test revealed that models violated the assumption of linearity of the logit; therefore, an age-squared term was included in these models. To overcome multicollinearity, the ORs and CIs are based on models using centred continuous variables. Controlling for age and sex only, having limited health literacy compared to adequate health literacy was associated with 49.3% greater odds of being a current smoker (model 1 OR = 1.493, 95% CI 1.307 to 1.704). A 1SD higher score in general cognitive ability was associated with 22.8% lower odds of reporting being a current smoker (model 2 OR = 0.772, 95% CI 0.718 to 0.829). Including both health literacy and general cognitive ability in model 3 reduced the size of the associations, but they remained significant. These associations continued to remain significant, though further attenuated, in the fully adjusted model, which additionally adjusted for age completed full-time education and occupational social class (model 4 OR for limited compared to adequate health literacy = 1.194, 95% CI 1.034 to 1.378; OR for a 1SD higher score in general cognitive ability =

For peer review only - http://bmjopen.bmj.com/site/about/guidelines.xhtml

0.878, 95% CI 0.810 to 0.951). In this final model, age left full-time education and occupational social class were also significantly associated with reporting being a current smoker. Compared to individuals who left full-time education at 14 years or under, those who left at age 17-18 or over 19 years had reduced odds of being a current smoker. Compared to those with a managerial or professional occupational class, those with a routine or manual occupational class had increased odds of being a current smoker.

DISCUSSION

This study found that in a sample of middle-aged and older adults residing in England, health literacy and cognitive ability were independently related with whether ever smokers continue to smoke nowadays, but not with whether individuals have ever smoked. Adjusting for age and sex only, participants with limited health literacy and lower cognitive ability were more likely to report having ever smoked. However, when additionally adjusting for age left full-time education and occupational class, these associations were attenuated and became non-significant. This suggests that health literacy and cognitive function do not have associations with ever smoking that are independent of education and occupational class. In ever smokers, those with limited health literacy and poorer cognitive ability were more likely to report that they continued to smoke. These associations remained, though slightly attenuated, even after adjusting for measures of socioeconomic status.

Whereas previous studies have found associations between health literacy and smoking,[13, 14] and cognitive ability and smoking,[3-10, 12] to the best of our knowledge, this is the first study to find that both health literacy and cognitive function are independently associated with smoking cessation. Health literacy and cognitive function are strongly related[27-30] and some have proposed that health literacy is not a unique construct and is, rather, a

For peer review only - http://bmjopen.bmj.com/site/about/guidelines.xhtml

subcomponent of general cognitive ability.[30] The current study, however, found that both health literacy and cognitive ability each play independent roles in predicting smoking cessation which is in support of health literacy and cognitive ability being separate, albeit related, constructs.

A particularly important finding from the current study was that health literacy, independent of cognitive ability, education and occupational social class, was associated with whether ever smokers continued to smoke. Health literacy, unlike already-established measures of socioeconomic status and perhaps more so than cognitive ability, is potentially modifiable.[31] Health literacy is thought to be the complex set of skills that are required to navigate all aspects of health care,[17, 18] and include reading and numeracy skills, as well as health-related knowledge.[32] At least one component of health literacy—health knowledge—may be increased through educational programs and interventions[32] and this in turn could lead to improved health outcomes.[33] Future research should examine whether cognitive ability and health literacy play a role in the success of smoking interventions, and should investigate whether interventions designed to increase smoking-specific health knowledge increase smoking cessation in individuals with limited health literacy.

This cross-sectional study was interested in examining the characteristics of smokers and, although a relationship between cognitive ability, health literacy and smoking cessation was identified, this study cannot determine the directionality of this association. It is possible that individuals who continue to smoke have lower cognitive ability and health literacy because smoking has damaging effects on both health literacy and cognitive ability. It is also possible that individuals who have lower cognitive ability and are less health literate are more likely to continue smoking because they do not have the cognitive capacity or health-related knowledge and skills required to fully comprehend the adverse effects of continuing to smoke on health, or the knowledge and skills required to access and use smoking cessation services.

For peer review only - http://bmjopen.bmj.com/site/about/guidelines.xhtml

For cognitive ability, evidence suggests that both of these pathways may be at least partially correct. Individuals with higher cognitive ability early in life are less likely to start smoking and more likely to quit,[10, 12] and smoking may cause steeper cognitive change throughout life.[4-7] A similar relationship may exist between health literacy and smoking. Further longitudinal studies which include measures of cognitive ability and health literacy in early life are needed to understand the pathways between health literacy, cognitive ability and smoking.

The key strengths of this study include the large sample size and the fact that ELSA was designed to be representative of individuals aged over 50 residing in England.[19] One limitation is that smoking status was self-reported; however, self-reported smoking has been found to be in agreement with serum cotinine measurements.[34] The smoking measures used here do not take into account the amount smoked throughout life. These results reported here may underestimate the true effect sizes because lifetime smoking was not considered.

Another limitation of this study is that the cognitive ability and health literacy tests used here were brief. Health literacy was assessed using a four-item test that was relatively insensitive to individual differences. That is, most individuals (67.3%) answered all questions correctly. Many, more detailed, health literacy assessments are available, such as the Test of Functional Health Literacy in Adults[35, 36] which is thought to be the gold-standard measure of health literacy.[37] More detailed health literacy tests may be more sensitive to detecting associations between health literacy and health. However, the brief four-item measure of health literacy used in ELSA has been found to be associated with mortality[21] and participation in cancer screening,[22] suggesting it is sensitive enough to detect associations with health.

For peer review only - http://bmjopen.bmj.com/site/about/guidelines.xhtml

The measure of general cognitive ability created here was constructed using a small number of brief cognitive tests that did not include, for example, reasoning that is highly loaded on general cognitive ability.[38] A better general cognitive ability measure would have been possible had more domains of cognitive function been assessed, with more detailed tests. Given other studies which have suggested that some health literacy measures are essentially aspects of cognitive function,[30, 39] and given also the limited cognitive test battery used in ELSA, it is possible that some of the independent contribution of the health literacy measure here is residual cognitive capability not picked up by the limited general cognitive ability component.

In this study of middle-aged and older adults, lower cognitive ability and poorer health literacy were associated with whether ever smokers continued to smoke, even after adjusting for education and occupational class. Further research is needed to identify possible pathways between health literacy, cognitive function and starting and quitting smoking.

For peer review only

Acknowledgements We are grateful to the ELSA participants, and to the UK Data Archive for supplying the data.

Contributors CFR discussed and planned the study and analyses, analysed the data, interpreted the data, and drafted the initial manuscript. JMS discussed and planned the study and analysis, interpreted the data, and contributed to the manuscript. IJD discussed and planned the study and analysis, interpreted the data, and contributed to the manuscript.

Funding This work was supported by the University of Edinburgh Centre for Cognitive Ageing and Cognitive Epidemiology, part of the cross council Lifelong Health and Wellbeing Initiative, funded by the Biotechnology and Biological Sciences Research Council (BBSRC), and Medical Research Council (MRC) (grant number MR/K026992/1).

Ethical approval Ethical approval for the English Longitudinal Study of Ageing was obtained from the National Research and Ethics Committee.

Competing interests None declared.

Data sharing statement No additional data available. Anonymised data from the English Longitudinal Study of Ageing is available from the UK Data Service (https://www.ukdataservice.ac.uk/).

For peer review only

For peer review only - http://bmjopen.bmj.com/site/about/guidelines.xhtml

REFERENCES

1 Office for National Statistics. Adult smoking habits in the UK: 2016. Retrieved from: https://www.ons.gov.uk/peoplepopulationandcommunity/healthandsocialcare/healthandlifeexpectancies/bulletins/adultsmokinghabitsingreatbritain/2016. England: Public Health England 2017 (accessed January 2018).

2 Allender S, Balakrishnan R, Scarborough P, et al. The burden of smoking-related ill health in the UK. *Tob Control* 2009;18(4):262-7. doi:10.1136/tc.2008.026294 [published Online First: 9 June 2009].

3 Mons U, Schottker B, Muller H, et al. History of lifetime smoking, smoking cessation and cognitive function in the elderly population. *Eur J Epidemiol* 2013;28(10):823-31. doi:10.1007/s10654-013-9840-9 [published Online First: 30 August 2013].

4 Reitz C, Luchsinger J, Tang MX, et al. Effect of smoking and time on cognitive function in the elderly without dementia. *Neurology* 2005;65(6):870-5. doi:10.1212/01.wnl.0000176057.22827.b7 [published Online First: 26 September 2005].

5 Richards M, Jarvis MJ, Thompson N, et al. Cigarette smoking and cognitive decline in midlife: evidence from a prospective birth cohort study. *Am J Public Health* 2003;93(6):994-8. doi:10.2105/AJPH.93.6.994.

6 Anstey KJ, von Sanden C, Salim A, et al. Smoking as a risk factor for dementia and cognitive decline: a meta-analysis of prospective studies. *Am J Epidemiol* 2007;166(4):367-78. doi:10.1093/aje/kwm116[published Online First: 14 June 2007].

7 Deary IJ, Pattie A, Taylor MD, et al. Smoking and cognitive change from age 11 to age 80. *J Neurol Neurosurg Psychiatry* 2003;74(7):1006-7. doi:10.1136/jnnp.74.7.1006.

8 Peters R, Poulter R, Warner J, et al. Smoking, dementia and cognitive decline in the elderly, a systematic review. *BMC Geriatr* 2008;8:36. doi:10.1186/1471-2318-8-36.

For peer review only

For peer review only - http://bmjopen.bmj.com/site/about/guidelines.xhtml

9 Corley J, Gow AJ, Starr JM, et al. Smoking, childhood IQ, and cognitive function in old age. *J Psychosom Res* 2012;73(2):132-8. doi:10.1016/j.jpsychores.2012.03.006 [published Online First: 26 April 2012].

10 Batty GD, Deary IJ, Schoon I, et al. Mental ability across childhood in relation to risk factors for premature mortality in adult life: the 1970 British Cohort Study. *J Epidemiol Community Health* 2007;61(11):997-1003. doi:10.1136/jech.2006.054494.

11 Hemmingsson T, Kriebel D, Melin B, et al. How does IQ affect onset of smoking and cessation of smoking-linking the Swedish 1969 conscription cohort to the Swedish survey of living conditions. *Psychosom Med* 2008;70(7):805-10. doi:10.1097/PSY.0b013e31817b955f [published Online First: 7 July 2008].

12 Taylor MD, Hart CL, Davey Smith G, et al. Childhood mental ability and smoking cessation in adulthood: prospective observational study linking the Scottish Mental Survey 1932 and the Midspan studies. *J Epidemiol Community Health* 2003;57(6):464-5. doi:10.1136/jech.57.6.464.

13 Fernandez DM, Larson JL, Zikmund-Fisher BJ. Associations between health literacy and preventive health behaviors among older adults: findings from the health and retirement study. *BMC Public Health* 2016;16:596. doi:10.1186/s12889-016-3267-7.

14 von Wagner C, Knight K, Steptoe A, et al. Functional health literacy and health-promoting behaviour in a national sample of British adults. *J Epidemiol Community Health* 2007;61(12):1086-90. doi:10.1136/jech.2006.053967.

15 Geboers B, Reijneveld SA, Jansen CJ, et al. Health Literacy Is Associated With Health Behaviors and Social Factors Among Older Adults: Results from the LifeLines Cohort Study. *J Health Commun* 2016;21(sup2):45-53. doi:10.1080/10810730.2016.1201174 [published Online First: 23 September 2016].

For peer review only

For peer review only - http://bmjopen.bmj.com/site/about/guidelines.xhtml

16 Wolf MS, Gazmararian JA, Baker DW. Health literacy and health risk behaviors among older adults. *Am J Prev Med* 2007;32(1):19-24. doi:10.1016/j.amepre.2006.08.024 [published Online First: 20 Dec 2006].

17 Institute of Medicine. Health Literacy: A Prescription to End Confusion. Washington DC: National Academies Press 2004.

18 Baker DW. The meaning and the measure of health literacy. *J Gen Intern Med* 2006;21(8):878-83. doi:10.1111/j.1525-1497.2006.00540.x.

19 Steptoe A, Breeze E, Banks J, et al. Cohort profile: the English longitudinal study of ageing. *Int J Epidemiol* 2013;42(6):1640-8. doi:10.1093/ije/dys168 [published Online First: 9 November 2012].

20 Thorn W. International Adult Literacy and Basic Skills Surveys in the OECD Regresion. OECD Educatio Working Papers. Paris: OECD Publishing 2009.

21 Bostock S, Steptoe A. Association between low functional health literacy and mortality in older adults: longitudinal cohort study. *BMJ* 2012;344:e1602. doi:10.1136/bmj.e1602.

22 Kobayashi LC, Wardle J, von Wagner C. Limited health literacy is a barrier to colorectal cancer screening in England: evidence from the English Longitudinal Study of Ageing. *Prev Med* 2014;61:100-5. doi:10.1016/j.ypmed.2013.11.012 [published Online First: 25 November 2013].

23 Gale CR, Deary IJ, Wardle J, et al. Cognitive ability and personality as predictors of participation in a national colorectal cancer screening programme: the English Longitudinal Study of Ageing. *J Epidemiol Community Health* 2015;69(6):530. doi:10.1136/jech-2014-204888 [published Online First: 3 February 2015].

24 Salthouse TA. Selective review of cognitive aging. *J Int Neuropsychol Soc* 2010;16(5):754-60. doi:10.1017/s1355617710000706 [published Online First: 2 August 2010].

For peer review only - http://bmjopen.bmj.com/site/about/guidelines.xhtml

25 Park DC, Reuter-Lorenz P. The adaptive brain: aging and neurocognitive scaffolding. *Annu Rev Psychol* 2009;60:173-96. doi:10.1146/annurev.psych.59.103006.093656.

26 Rose D, Pevalin DJ, O'Reilly K. The National Statistics Socio-economic Classification: origins, development and use. Basingstoke, UK: Palgrave Macmillan 2005.

27 Federman AD, Sano M, Wolf MS, et al. Health literacy and cognitive performance in older adults. *J Am Geriatr Soc* 2009;57(8):1475-80. doi:10.1111/j.1532-5415.2009.02347.x [published Online First: 8 June 2009].

28 Kobayashi LC, Wardle J, Wolf MS, et al. Cognitive Function and Health Literacy Decline in a Cohort of Aging English Adults. *J Gen Intern Med* 2015;30(7):958-64. doi:10.1007/s11606-015-3206-9 [published Online First: 14 February 2015].

29 Murray C, Johnson W, Wolf MS, et al. The Association between Cognitive Ability across the Lifespan and Health Literacy in Old Age: The Lothian Birth Cohort 1936. *Intelligence* 2011;39(4):178-87. doi:10.1016/j.intell.2011.04.001 [published Online First: 4 May 2011].

30 Reeve CL, Basalik D. Is health literacy an example of construct proliferation? A conceptual and empirical evaluation of its redundancy with general cognitive ability. *Intelligence* 2014;44:93-102. doi:10.1016/j.intell.2014.03.004 [published Online First: 16 April 2014].

31 Nutbeam D. The evolving concept of health literacy. *Soc Sci Med* 2008;67(12):2072-8. doi:10.1016/j.socscimed.2008.09.050 [published Online First: 25 October 2008].

32 Chin J, Morrow DG, Stine-Morrow EA, et al. The process-knowledge model of health literacy: evidence from a componential analysis of two commonly used measures. *J Health Commun* 2011;16 Suppl 3:222-41. doi:10.1080/10810730.2011.604702.

33 Rothman RL, DeWalt DA, Malone R, et al. Influence of patient literacy on the effectiveness of a primary care-based diabetes disease management program. *JAMA* 2004;292(14):1711-6. doi:10.1001/jama.292.14.1711.

For peer review only

For peer review only - http://bmjopen.bmj.com/site/about/guidelines.xhtml

34 Vartiainen E, Seppala T, Lillsunde P, et al. Validation of self reported smoking by serum cotinine measurement in a community-based study. *J Epidemiol Community Health* 2002;56(3):167-70. doi:10.1136/jech.56.3.167.

35 Parker RM, Baker DW, Williams MV, Nurss JR. The test of functional health literacy in adults: a new instrument for measuring patients' literacy skills. *J Gen Intern Med* 1995;10(10):537-41.

36 Baker DW, Williams MV, Parker RM, Gazmararian JA, Nurss J. Development of a brief test to measure functional health literacy. *Patient Educ Couns* 1999;38(1):33-42. doi: 10.1016/S0738-3991(98)00116-5.

37 Mancuso JM. Assessment and measurement of health literacy: an integrative review of the literature. *Nurs Health Sci 2009*;11(1):77-89. doi: 10.1111/j.1442-2018.2008.00408.x.

38 Salthouse TA. Localizing age-related individual differences in a hierarchical structure. *Intelligence* 2004;32(6). doi:10.1016/j.intell.2004.07.003 [published Online First: 11 September 2004].

39 Mottus R, Johnson W, Murray C, et al. Towards understanding the links between health literacy and physical health. *Health Psychology* 2014;33(2):164-73. doi:10.1037/a0031439.

For peer review only

For peer review only - http://bmjopen.bmj.com/site/about/guidelines.xhtml

STROBE 2007 (v4) Statement—Checklist of items that should be included in reports of *cohort studies*

Section/Topic	Item #	Recommendation	Reported on page #
**Title and abstract**	1	(*a*) Indicate the study's design with a commonly used term in the title or the abstract	1
		(*b*) Provide in the abstract an informative and balanced summary of what was done and what was found	2
**Introduction**			
Background/rationale	2	Explain the scientific background and rationale for the investigation being reported	3-4
Objectives	3	State specific objectives, including any prespecified hypotheses	4
**Methods**			
Study design	4	Present key elements of study design early in the paper	4-7
Setting	5	Describe the setting, locations, and relevant dates, including periods of recruitment, exposure, follow-up, and data collection	4-5
Participants	6	(*a*) Give the eligibility criteria, and the sources and methods of selection of participants. Describe methods of follow-up	4-5
		(*b*) For matched studies, give matching criteria and number of exposed and unexposed	NA
Variables	7	Clearly define all outcomes, exposures, predictors, potential confounders, and effect modifiers. Give diagnostic criteria, if applicable	5-7
Data sources/ measurement	8*	For each variable of interest, give sources of data and details of methods of assessment (measurement). Describe comparability of assessment methods if there is more than one group	4-7
Bias	9	Describe any efforts to address potential sources of bias	NA
Study size	10	Explain how the study size was arrived at	4-5, 8
Quantitative variables	11	Explain how quantitative variables were handled in the analyses. If applicable, describe which groupings were chosen and why	7
Statistical methods	12	(*a*) Describe all statistical methods, including those used to control for confounding	7
		(*b*) Describe any methods used to examine subgroups and interactions	7
		(*c*) Explain how missing data were addressed	8
		(*d*) If applicable, explain how loss to follow-up was addressed	NA
		(*e*) Describe any sensitivity analyses	NA
**Results**			

For peer review only

Participants	13*	(a) Report numbers of individuals at each stage of study—eg numbers potentially eligible, examined for eligibility, confirmed eligible, included in the study, completing follow-up, and analysed	4-5, 8
		(b) Give reasons for non-participation at each stage	4-5, 8
		(c) Consider use of a flow diagram	NA
Descriptive data	14*	(a) Give characteristics of study participants (eg demographic, clinical, social) and information on exposures and potential confounders	8, 10-11
		(b) Indicate number of participants with missing data for each variable of interest	10-11
		(c) Summarise follow-up time (eg, average and total amount)	NA
Outcome data	15*	Report numbers of outcome events or summary measures over time	8, 10-11
Main results	16	(*a*) Give unadjusted estimates and, if applicable, confounder-adjusted estimates and their precision (eg, 95% confidence interval). Make clear which confounders were adjusted for and why they were included	12-15
		(*b*) Report category boundaries when continuous variables were categorized	NA
		(*c*) If relevant, consider translating estimates of relative risk into absolute risk for a meaningful time period	NA
Other analyses	17	Report other analyses done—eg analyses of subgroups and interactions, and sensitivity analyses	NA
**Discussion**			
Key results	18	Summarise key results with reference to study objectives	16
**Limitations**			
Interpretation	20	Give a cautious overall interpretation of results considering objectives, limitations, multiplicity of analyses, results from similar studies, and other relevant evidence	16-18
Generalisability	21	Discuss the generalisability (external validity) of the study results	17-18
**Other information**			
Funding	22	Give the source of funding and the role of the funders for the present study and, if applicable, for the original study on which the present article is based	19

*Give information separately for cases and controls in case-control studies and, if applicable, for exposed and unexposed groups in cohort and cross-sectional studies.

Note: An Explanation and Elaboration article discusses each checklist item and gives methodological background and published examples of transparent reporting. The STROBE checklist is best used in conjunction with this article (freely available on the Web sites of PLoS Medicine at http://www.plosmedicine.org/, Annals of Internal Medicine at http://www.annals.org/, and Epidemiology at http://www.epidem.com/). Information on the STROBE Initiative is available at www.strobe-statement.org.

For peer review only
